# Supplementary material for: Local Response and Barrier Recovery in Elderly Skin Following the Application of High-Density Microarray Patches
Source: Vaccines (Basel). 2022 Apr 10;10(4):583. doi: 10.3390/vaccines10040583 (PMC9031416; doi:10.3390/vaccines10040583)
Supplement: Supplementary file 1 [file vaccines-10-00583-s001.zip › vaccines-1642284-supplementary.pdf]

Supplementary Materials, Table S1. Demographics.

| **S1A, Forearm** | | | | | | | **Bleeding** | **Pain (0-10)** | | **Bruising Yes/No** | | **Hypopigmentation** | **Hyperpigmentation** |
|---|---|---|---|---|---|---|---|---|---|---|---|---|---|
| **Subject** | **Sex** | **Age (years)** | **Height (cm)** | **Weight (kg)** | **BMI (kg/m²)** | **Arm** | **Yes/No** | **<10 min** | **30 min** | **<10 min** | **30 min** | **48 hrs Yes/No** | **48 hrs Yes/No** |
| 401 | M | 77 | 178 | 66 | 21 | L | Y | 0 | 0 | N | N | N | N |
| 402 | M | 84 | 168 | 95 | 34 | L | Y | 0 | 0 | N | N | N | N |
| 403 | M | 73 | 172 | 80 | 27 | L | N | 0 | 0 | N | N | N | N |
| 404 | M | 83 | 175 | 76 | 25 | L | Y | 1 | 0 | N | N | N | N |
| 405 | M | 71 | 178 | 79 | 25 | R | N | 3 | 0 | N | N | N | N |
| 406 | M | 79 | 182 | 90 | 27 | R | N | 1 | 0 | N | N | N | N |
| 407 | F | 74 | 165 | 75 | 28 | R | Y | 0 | 2 | N | N | N | N |
| 408 | F | 75 | 172 | 79 | 27 | R | N | 3 | 0 | N | N | N | N |
| 409 | M | 73 | 170 | 75 | 26 | R | Y | 2 | 0 | Y | N | N | N |
| 410 | M | 69 | 180 | 120 | 37 | L | Y | 0 | 0 | Y | N | N | N |
| 411 | F | 71 | 153 | 57 | 24 | L | N | 3 | 0 | Y | N | N | N |
| 412 | M | 74 | 172 | 62 | 21 | R | Y | 2 | 0 | Y | N | N | N |
| **Mean (SD)** | | 75±5 | 172±8 | 80±17 | 27±5 | | | | | | | | |

| S1B, Deltoid Subject | Sex | Age (years) | Height (cm) | Weight (kg) | BMI (kg/m$^2$) | Arm | Bleeding Yes/No | Pain (0-10) <10 min | Pain (0-10) 30 min | Bruising Yes/No <10 min | Bruising Yes/No 30 min | Hypopigmentation 48 hrs Yes/No | Hyperpigmentation 48 hrs Yes/No |
|---|---|---|---|---|---|---|---|---|---|---|---|---|---|
| 401 | M | 77 | 178 | 66 | 21 | L | Y | 0 | 0 | N | N | N | N |
| 402 | M | 84 | 168 | 95 | 34 | L | N | 0 | 0 | N | N | N | N |
| 403 | M | 73 | 172 | 80 | 27 | L | N | 0 | 0 | N | N | N | N |
| 404 | M | 83 | 175 | 76 | 25 | L | N | 1 | 0 | N | N | N | N |
| 405 | M | 71 | 178 | 79 | 25 | R | N | 2 | 0 | N | N | N | N |
| 406 | M | 79 | 182 | 90 | 27 | R | N | 1 | 0 | N | N | N | N |
| 407 | F | 74 | 165 | 75 | 28 | R | Y | 2 | 0 | N | N | N | N |
| 408 | F | 75 | 172 | 79 | 27 | R | Y | 3 | 0 | N | N | N | N |
| 409 | M | 73 | 170 | 75 | 26 | R | N | 2 | 0 | N | N | N | N |
| 410 | M | 69 | 180 | 120 | 37 | L | N | 0 | 0 | N | N | N | N |
| 411 | F | 71 | 153 | 57 | 24 | L | Y | 2 | 0 | N | N | N | N |
| 412 | M | 74 | 172 | 62 | 21 | R | Y | 2 | 0 | N | N | N | N |
| **Mean (SD)** | | 75±5 | 172±8 | 80±17 | 27±5 | | | | | | | | |
